# Microbiome Differences in Preeclampsia Versus Lupus Nephritis

**DOI:** 10.7759/cureus.101296

**Published:** 2026-01-11

**Authors:** Harshita Nadella, Julia L Armstrong, Rachel Greenwald, Kenneth Johnson, Marc M Kesselman

**Affiliations:** 1 Rheumatology and Immunology, Nova Southeastern University Dr. Kiran C. Patel College of Osteopathic Medicine, Davie, USA; 2 Internal Medicine, Nova Southeastern University Dr. Kiran C. Patel College of Osteopathic Medicine, Davie, USA; 3 Obstetrics and Gynecology, Nova Southeastern University Dr. Kiran C. Patel College of Osteopathic Medicine, Davie, USA; 4 Rheumatology, Nova Southeastern University Dr. Kiran C. Patel College of Osteopathic Medicine, Davie, USA

**Keywords:** high-risk pregnancy, hypertensive states of pregnancy, lupus nephritis flare, pre-eclampsia, sle pathogenesis

## Abstract

Preeclampsia (PE) and lupus nephritis (LN) share clinical features of hypertension, proteinuria, and systemic inflammation, reflecting overlapping immune dysregulation. Both conditions involve activation of pro-inflammatory cytokines and endothelial dysfunction, which contribute to organ damage. They also exhibit similarities in their microbiomes, including reduced diversity and loss of beneficial immunoregulatory taxa, which may exacerbate systemic inflammation. Despite similarities, they differ in etiology. PE results from placental dysfunction, whereas LN arises from autoimmune-driven renal injury. PE is associated with enrichment of pro-inflammatory microbes, which contribute to endothelial dysfunction and impaired trophoblast invasion. In contrast, LN exhibits gut dysbiosis involving expansion of pro-inflammatory species and depletion of protective immunoregulatory taxa, promoting intestinal permeability and renal inflammation. These shared, disease-specific microbiome features suggest potential for diagnostic differentiation and help guide future microbiome-targeted therapies.

## Introduction and background

The purpose of this review is to identify whether microbiome profiles can distinguish preeclampsia (PE) from lupus nephritis (LN). To accomplish this, we compare microbiome differences between PE and LN across multiple body sites, including the gut, vaginal, and placental compartments. Drawing on high-quality literature published since 2020, we aim to identify distinct and shared microbiomes, explore how these relate to immune and vascular dysfunction, and evaluate their potential utility as diagnostic biomarkers, risk stratifiers, and therapeutic targets.

Hypertensive pregnancy disorders occur in 6-8% of pregnancies in the United States. PE is part of a spectrum of hypertensive pregnancy disorders ranging from chronic hypertension to HELLP (hemolysis, elevated liver enzymes, low platelet count) syndrome, each defined by specific criteria and management (Table 1). Metabolic diseases such as obesity and diabetes, cocaine use, advanced maternal age, chronic kidney disease (CKD), and nulliparity may lead to endothelial dysfunction, impaired placental implantation, and increased risk for the development of hypertensive pregnancy disorders [1]. PE is a complex, multisystem hypertensive disorder that arises after 20 weeks’ gestation, marked by new-onset hypertension (≥140/90 mmHg) and proteinuria or end-organ dysfunction [1]. Affecting approximately 5-7% of all pregnancies in the United States, PE remains a leading cause of maternal and perinatal morbidity and mortality, particularly in low- and middle-income women [1,2].

**Table 1:** Hypertensive disorders of pregnancy: diagnostic criteria and management. ALT = alanine aminotransferase; AST = aspartate aminotransferase; BP = blood pressure; Cr = creatinine; Hb = hemoglobin; HTN = hypertension; LDH = lactate dehydrogenase; RUQ = right upper quadrant; ULN = upper limit of normal; US = ultrasound

Diagnosis	Diagnostic criteria	Management
Chronic hypertension	HTN diagnosed before pregnancy or in the first 20 weeks of pregnancy	Consider delivery at 37 weeks; lifestyle modifications; consider antihypertensives if BP ≥140/90
Gestational hypertension	New-onset HTN ≥140/90 after 20 weeks; no prior HTN; asymptomatic	Consider delivery at 37 weeks; lifestyle modifications; consider antihypertensives if BP is consistently ≥140/90
Preeclampsia (without severe features)	HTN (≥140/90) and proteinuria diagnosed by: 24-hour urine collection >300 mg/24 hours; urine protein/creatinine ratio >0.3; urine dipstick >2+ protein	Consider delivery at 37 weeks; lifestyle modifications; consider antihypertensives if BP is consistently ≥140/90; educate on warning signs and symptoms of preeclampsia
Preeclampsia (with severe features)	Gestational HTN plus ≥1: severe HTN (≥160/110); thrombocytopenia (<100,000); impaired renal function (serum Cr >1.1 or 2× baseline); elevated AST/ALT (>2× ULN) or severe RUQ/epigastric pain; pulmonary edema; new onset of headache unresponsive to medication or visual disturbances (blurred vision, scotoma)	Consider delivery at 34 weeks if mother and fetus are stable; earlier if maternal/fetal distress; consider termination if pregnancy is prior to fetal viability; lifestyle modifications; start antihypertensives if BP is consistently ≥140/90; magnesium sulfate for seizure prophylaxis; monitor BP, O_2_, and urine output; continuous fetal monitoring, US, and appropriate antenatal testing (e.g., biophysical profile)
HELLP syndrome	Preeclampsia plus all: Hemolysis (↓ Hb, ↓ haptoglobin, ↑ LDH, ↑ indirect bilirubin); elevated liver enzymes (AST/ALT >2× ULN); low platelets <100,000	Start antihypertensives if BP is consistently ≥140/90; magnesium sulfate for seizure prophylaxis; monitor BP, O_2_, and urine output; continuous fetal monitoring; consult hematology; administer blood products if needed; continuous fetal monitoring and deliver immediately
Eclampsia	New-onset seizures in a patient with preeclampsia	Start continuous fetal monitoring and immediate delivery; treat seizures; airway/oxygen support; magnesium sulfate (add additional medications if needed); monitor BP, O_2_, and urine output; start antihypertensives if BP is consistently ≥140/90

While historically considered a placental disorder, modern research highlights its multifactorial etiology, involving aberrant placentation, systemic endothelial dysfunction, chronic inflammation, and maladaptive maternal immune responses to the semi-allogeneic fetus [3,4]. At the center of PE pathogenesis is a failure of early trophoblast invasion and spiral artery remodeling, leading to placental hypoxia and ischemia [5]. This results in acute atherosis of the decidual vessels, characterized by arterial wall fibrinoid necrosis and lymphoid infiltration [5]. Due to the abnormal blood flow, hypertension with systemic vasoconstriction causes placental hypoperfusion. This hypoxic stress stimulates the release of antiangiogenic factors such as soluble fms-like tyrosine kinase-1 and soluble endoglin, which antagonize vascular endothelial growth factor and placental growth factor signaling, leading to maternal endothelial dysfunction and multisystem injury [5]. These pathological changes are further compounded by immune dysregulation, characterized by exaggerated natural killer cell activation, increased T-helper (Th)1/Th17 responses, and impaired regulatory T-cell function, all of which contribute to poor placental perfusion and systemic inflammation [6]. An increase in the placental factors of inflammation and dysfunction leads to endothelial lesions that result in microthrombosis [7]. There is an array of consequences from vasoconstriction and microthrombosis across a multitude of organs beyond the placenta, as illustrated in Table 2.

**Table 2:** Pathophysiology and organ-specific injury in preeclampsia. CNS = central nervous system; DIC = disseminated intravascular coagulation; SVR = systemic vascular resistance

Organ	Pathogenesis
Kidney	Glomerular endothelial dysfunction and hypertension-induced vasoconstriction lead to proteinuria, impaired renal function, and edema
Lung	Increased SVR and volume overload lead to left ventricular dysfunction, increased pulmonary capillary hydrostatic pressure, increased capillary permeability, and decreased albumin; this leads to pulmonary edema and respiratory distress
Liver	Vasoconstriction and microthrombotic obstruction lead to liver cell damage and liver swelling
CNS	Vasoconstriction and endothelial damage lead to vasospasm in the CNS, which can lead to seizures
Blood	Systemic microthrombi and vasoconstriction lead to overactivation of the coagulation system and platelet consumption, which leads to DIC, thrombocytopenia, and anemia

Systemic lupus erythematosus (SLE) is an autoimmune disease characterized by the production of autoantibodies and immune complex deposition. This leads to chronic systemic inflammation and multi-organ damage. Among its many manifestations, LN is one of the most serious and common complications, affecting up to 60% of patients with SLE at some point in their lives [8]. LN is a well-recognized cause of CKD and end-stage renal disease in young women, particularly among African Americans and Hispanics who exhibit higher prevalence and worse outcomes [9]. LN also disproportionately affects women of reproductive age and can both mimic or complicate the diagnosis of PE during pregnancy [9].

The pathogenesis of LN involves the formation of circulating immune complexes containing anti-double-stranded DNA (anti-dsDNA) antibodies and/or anti-Smith (anti-Sm) antibodies, which deposit in the mesangial or subendothelial regions of the glomerulus [8]. This triggers a local inflammatory response through complement activation and downstream cytokine release involving T cells, B cells, and kidney cells, ultimately resulting in varying degrees of glomerular injury, including thickening of the mesangium, capillary walls, and glomerular basement membrane [8]. This pathophysiology also shares several overlapping features with PE, including immune dysregulation, endothelial injury, and proteinuria [3,7]. The most common clinical manifestations of LN are hypertension, edema, and hematuria, which mirror those seen in PE. Diagnosis requires laboratory evaluation, including a complete blood count, basic metabolic panel (BMP), and urinalysis. The BMP typically reveals elevated creatinine, while the urinalysis may show proteinuria, hematuria, cellular casts, and a urine protein-to-creatinine ratio >0.5 [10]. A kidney biopsy is performed when indicated for the following reasons: an unexplained increase in creatinine, proteinuria >1.0 g/day, or proteinuria >0.5 g/day with hematuria or cellular urinary casts [10]. The biopsy findings demonstrate immune complex-mediated glomerulonephritis [10].

Histologically, LN is classified by the International Society of Nephrology/Renal Pathology Society into six distinct classes, ranging from minimal mesangial involvement to severe proliferative or sclerotic changes (Table 3). All patients with LN should be evaluated by a nephrologist, especially those in class III or IV [10]. These two classes typically require both induction and maintenance therapy. Induction therapy consists of intravenous (IV) glucocorticoids, such as methylprednisolone, and immunosuppressants, such as mycophenolate or cyclophosphamide [11]. Maintenance therapy includes oral prednisone and mycophenolate or azathioprine [11]. Despite advances in immunosuppressive regimens, including the use of corticosteroids, mycophenolate, and biologics such as belimumab and voclosporin, many patients do not achieve remission and may experience relapse [11]. Moreover, long-term immunosuppressant use carries substantial risks such as infection or renal and hepatic toxicity [11]. These challenges illustrate the urgent need for biomarkers and therapeutic targets that enable personalized treatment strategies, which emerging microbiome research might help guide. A renal biopsy is also used to confirm the class and thus guide therapy. Angiotensin-converting enzyme inhibitors and angiotensin II receptor blockers are also commonly used for the control of proteinuria and as first-line antihypertensive agents [8].

**Table 3:** International Society of Nephrology/Renal Pathology Society classification of lupus nephritis and corresponding histopathology.

Class	Histopathology
Class 1	Minimal mesangial lupus nephritis
Class 2	Mesangial proliferative lupus nephritis
Class 3	Focal lupus nephritis (<50% of glomeruli involved)
Class 4	Diffuse lupus nephritis (≥50% of glomeruli involved)
Class 5	Membranous lupus nephritis
Class 6	Advanced sclerosing lupus nephritis

The human microbiome refers to the vast collection of microorganisms, including bacteria, viruses, fungi, and archaea, that inhabit various anatomical sites of the body, such as the gut, skin, oral cavity, vagina, and urinary tract [12]. These microbial communities play an integral role in host immune function, metabolism, barrier integrity, and even neurocognitive development. Over the past two decades, advances in microbial profiling have allowed scientists to expand their understanding of the microbiome’s functional capacity. This has contributed to a paradigm shift in medicine, from viewing microbiomes solely as pathogens to recognizing them as critical modulators of health and disease across multiple organ systems.

Advances in sequencing technologies have revealed the microbiome’s involvement in a wide range of diseases, including inflammatory bowel disease, diabetes, cardiovascular disease, neuropsychiatric disorders, autoimmune conditions such as SLE, and reproductive complications such as PE [12]. Clinically, the microbiome is now used both diagnostically and therapeutically, with interventions such as fecal microbiota transplantation, probiotics, and dietary modulation in patients [12]. The evolving clinical applications of the microbiome extend beyond diagnostics. With the emergence of “precision microbiome editing, live biotherapeutics, and postbiotic interventions,” the microbiome is increasingly viewed as both a diagnostic biomarker and treatment target [13]. A myriad of trials are exploring microbiota modulation to improve outcomes in several pathologies such as sepsis, transplant medicine, and autoimmune diseases. With antibiotic resistance increasing and more adverse effects of biologic therapies coming to light, microbiome-based strategies may become a focus across many fields as potential treatment alternatives. As a result, understanding the microbiome’s compositional shifts and the resulting functional disruption in the host is vital, particularly in complex systemic conditions such as LN and PE, where the interplay between immunity, inflammation, and barrier dysfunction plays a central role in the patient’s health [13]. Despite extensive research on the microbiome in each disease separately, no comprehensive synthesis has compared their microbial profiles directly. This review aims to address this gap by evaluating and comparing the microbiota composition in SLE-LN and PE and identifying opportunities for diagnostic and therapeutic advancements.

## Review

Methodology

Study Design

An in-depth scoping review was conducted to compare microbiome alterations between patients with LN and those with PE. This review followed the Preferred Reporting Items for Systematic Reviews and Meta-Analyses (PRISMA) recommendations. Human observational studies were analyzed as the primary evidence base, while animal studies were included separately to provide mechanistic support and were not interpreted as direct clinical evidence. This scoping review aimed to capture the breadth of existing literature and therefore included a range of study designs, including human primary studies, animal studies, and review articles.

Search Strategy

A literature search was performed using PubMed as the primary database, limiting articles published between January 2020 and April 2025. To ensure validity, two separate searches were conducted, one for LN and one for PE. Search terms included “lupus nephritis” AND “microbiome” for LN, and “preeclampsia” AND "microbiome” for PE.

Study Selection Criteria

Eligible studies included those published in English between 2020 and 2025, accessible through institutional resources, and focused on human or animal models that investigated the association between LN or PE and microbiome composition. Study designs included animal model experiments, human observational cohorts, review articles, and systematic reviews. Exclusion criteria included studies focused on non-microbiome mechanisms, studies lacking a control group, studies not relevant to the research question, and case reports.

Study Layout

Most human studies analyzed fecal bacterial communities using 16S rRNA sequencing, with sample sizes ranging from 38 to 221. Additional analytical methods included shotgun metagenomic sequencing, qPCR, in situ hybridization, gas chromatography, and bioinformatics.

**Figure 1:**
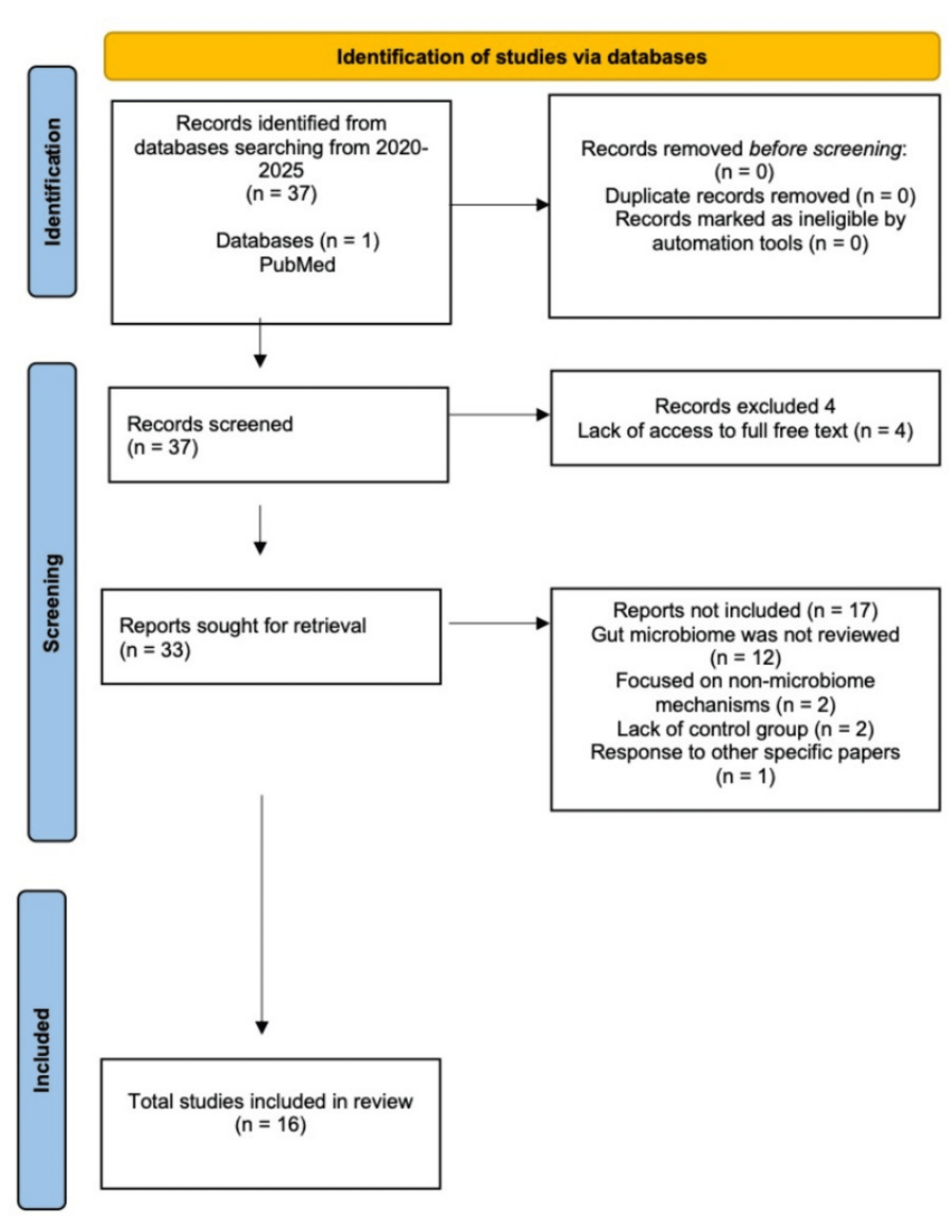
Preferred Reporting Items for Systematic Reviews and Meta-Analyses (PRISMA) flowchart for lupus nephritis microbiome alterations.

For LN, 37 records were identified. After screening, 33 full-text articles were reviewed, and 16 met the inclusion criteria for this review (Figure 1). Studies focusing primarily on skin, genital, urinary, or fungal microbiota were excluded from the primary comparative analysis due to the limited availability of studies directly linking these microbial sites to LN-specific outcomes. While genital microbiome changes have been described in PE, comparable controlled data in lupus nephritis remain limited, which restricts disease comparison.

**Figure 2:**
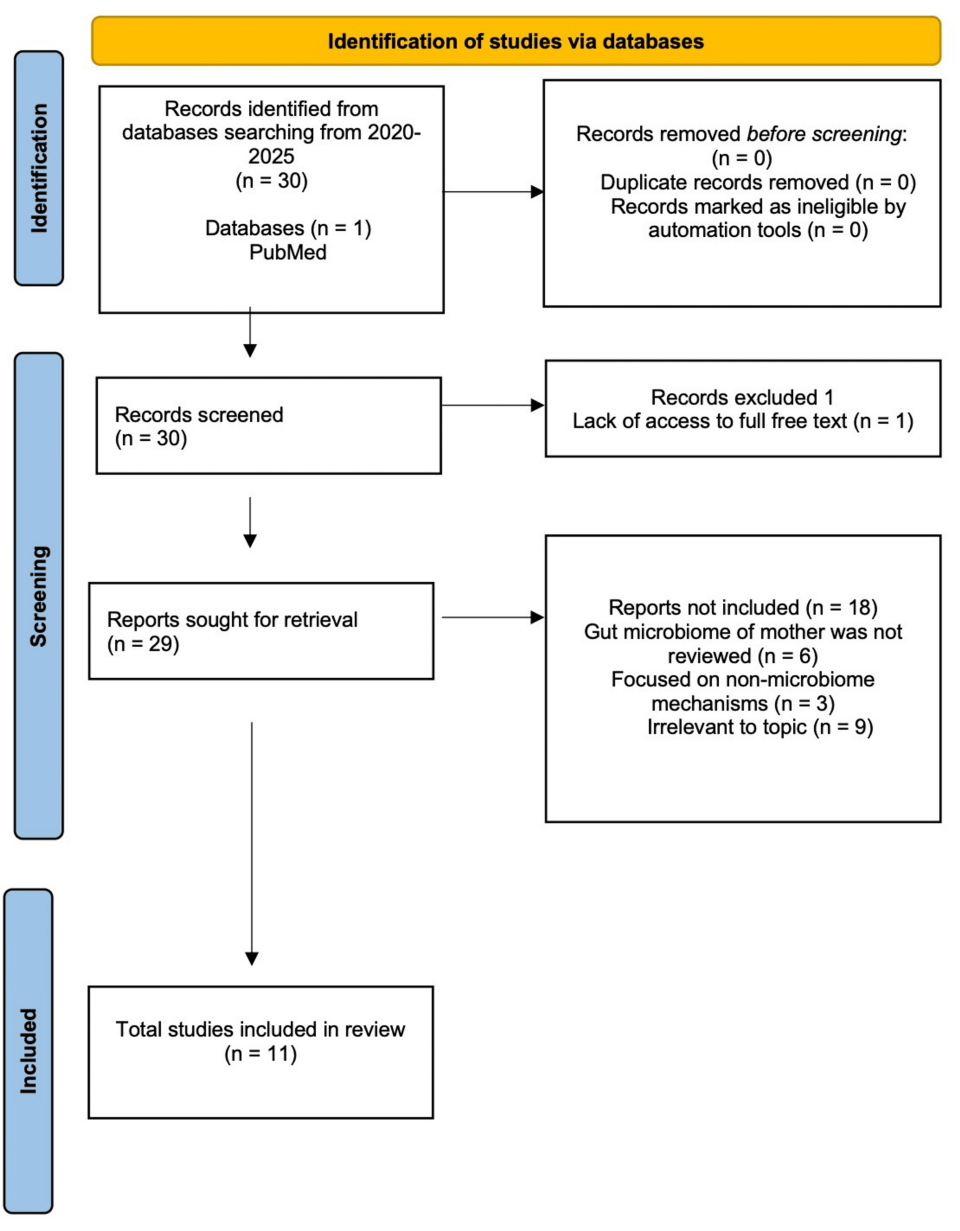
Preferred Reporting Items for Systematic Reviews and Meta-Analyses (PRISMA) flowchart for preeclampsia microbiome alterations.

For PE, a total of 30 articles examining the microbiome alterations were retrieved. One article was excluded due to a lack of full-text access, leaving 29 articles for screening. Screening was performed by reviewing abstracts and removing out-of-scope articles, followed by full-text analyses. After screening, 11 articles met all inclusion criteria and were included in this review (Figure 2).

Data Extraction

Data extraction was performed independently by three reviewers and included study type, population type, analysis method, sample size, and key findings. Google Docs was implemented to organize article reviews for improved extraction and analysis of overarching themes. Once finalized, EndNote was used to manage citations and references. No additional unpublished data were requested from the original study authors.

Results

Lupus Nephritis: Microbiome Findings

The phylum Proteobacteria was reported to be elevated in multiple studies, including Cheng et al., Wang et al., Parodi et al., Wu et al., and Yu et al. [14-18]. Expansions of *Ruminococcus gnavus* *(R. gnavus)* were frequently noted during LN flares or in patients with more severe disease [19,20]. Supporting this, Silverman et al. demonstrated that *R. gnavus* strains isolated from LN patients could trigger gut barrier dysfunction and activate the immune system in lupus-prone mice, suggesting these bacteria may play a direct role in worsening disease [21]. Additionally, Gui et al. reported that overgrowth of *Escherichia*, particularly *Escherichia coli*, was enriched in LN patients and associated with greater disease severity [22]. In this study, researchers also found that fecal transfer of these bacteria worsened kidney damage and immune activation in lupus-prone mice [22]. Similarly, Cheng et al. found significant enrichment of *Enterobacteriaceae*, *Escherichia-Shigella*, and *E. coli *among LN patients, alongside reduced abundance of *Faecalibacterium* and Clostridia [18]. Among systematic reviews, Wang et al. confirmed consistent reductions in diversity and the Firmicutes/Bacteroidetes (F/B) ratio in LN, along with enrichment of Proteobacteria and specific taxa such as *R. gnavus* and *Bacteroides thetaiotaomicron* [16]. However, Tan et al. noted that the F/B ratios in SLE and LN are not consistent across all studies [23]. Gui et al. found an increased Proteobacteria-to-Bacteroidetes (P/B) ratio in LN patients [22]. An additional study found that decreases in Firmicutes and the F/B ratio were not statistically significant [15].

In addition to consistent taxonomic shifts across LN patient studies, animal models also support a link between gut microbial alterations and disease severity. A study comparing genetically identical MRL/lpr mice (a lupus-prone murine model with a *Fas* gene mutation) from two different facilities reported significant differences in alpha diversity as measured by the Shannon Index (SI), along with distinct microbial compositions that correlated with differences in disease severity [24]. Mice from one colony developed more severe glomerulonephritis, which was associated with a higher abundance of Lactobacillaceae, whereas the other colony showed enrichment of Lachnospiraceae [24]. Further evidence for the role of gut dysbiosis in LN comes from studies of mesenchymal stem cell (MSC) therapy. Early MSC transplantation in MRL/lpr mice increased gut microbial diversity and was associated with improved kidney outcomes and enrichment of potentially beneficial strains such as *Lactobacillus johnsonii* and *Romboutsia ilealis* [25]. Gao et al. similarly demonstrated that fecal microbiota transplantation from healthy donors into lupus-prone mice led to expansion of *Lactobacillus johnsonii* and the metabolite inosine [25]. In another study, oral administration of *Bacteroides fragilis* to lupus-prone MRL/lpr mice reduced kidney pathology and serum autoantibody levels compared to untreated controls [26]. Fecal microbiota transplantation is presented only as an experimental approach in animal studies to support mechanistic interpretation and is not intended to suggest clinical relevance or therapeutic use. A summary of microbiome findings in human studies and animal studies is provided in Table 4 and Table 5.

**Table 4:** Summary of microbiome findings in lupus nephritis animal models. ATCC = American Type Culture Collection; BHI = brain–heart infusion; FMT = fecal microbiota transplantation; F/B ratio = Firmicutes-to-Bacteroidetes ratio; GF = germ-free; hUC-MSC = human umbilical cord–derived mesenchymal stem cells; JAX = The Jackson Laboratory; MSC = mesenchymal stem cells; MRL/lpr = lupus-prone mouse strain with *Fas* mutation; PBS = phosphate-buffered saline; PCR = polymerase chain reaction; qPCR = quantitative PCR; RG = *Ruminococcus gnavus*; sp. = species (singular); C57BL/6 = inbred laboratory mouse strain; C57BL/6J = substrain of C57BL/6; WGS = whole-genome sequencing; 16S rDNA = 16S ribosomal DNA; 16S rRNA = 16S ribosomal RNA; *B. fragilis* = *Bacteroides fragilis*; CAG-267 = *Acetobacter* strain identifier

Author (year)	Experimental group	Control group/Baseline	Microbiome source	Detection methods	Micro-organisms increased	Micro-organisms decreased
Gao et al. (2025) [25]	MRL/lpr mice at 10, 15, and 20 weeks	MRL/lpr mice at 4 weeks	Fecal pellets	16S rDNA sequencing; metagenome sequencing	Phylum: *Desulfobacterota;*Genus: *Bacteroides, Desulfovibrio;*Species: *Escherichia coli*	Phylum: Firmicutes; Genus: *Alistipes, Lactobacillus, Rikenella,* Clostridia UCG-014; Species: *Lactobacillus johnsonii, Lactobacillus murinus*
Silverman et al. (2022) [21]	C57BL/6 GF mice colonized with Lupus-derived *Blautia (Ruminococcus) gnavus* strains (S47-18, S107-48)	C57BL/6 GF mice colonized with healthy RG strain (RG1/ATCC29149)	Fecal samples (lupus nephritis patients S47/S107 for experimental strains; healthy donor for control strain)	Bacterial culture (BHI media plates); 16S rRNA-specific PCR amplification; whole-genome sequencing; RG-specific qPCR	Species: *Ruminococcus gnavus* in C57BL/6 GF mice colonized with Lupus-derived *Blautia (Ruminococcus) gnavus* strains	N/A
Li et al. (2020) [26]	MRL/lpr mice treated with *B. fragilis*	PBS-treated MRL/lpr mice (vehicle group); C57BL/6J mice (normal control)	Gut	N/A (no profiling; *B. fragilis* cultured and administered)	N/A	N/A
Pan et al. (2025) [27]	MRL/lpr mice: MSC (hUC-MSC at weeks 6, 8, 10); MSC-FMT (MSC + FMT at weeks 9–13)	MRL/lpr mice treated with saline	Fecal samples from 15-week-old mice	Metagenomic sequencing	Genus: *Romboutsia, Acetobacter, Coprobacillus* (trend); Species: *Romboutsia ilealis, Romboutsia timonensis, Romboutsia hominis, Romboutsia lituseburensis, Lactobacillus johnsonii, Ligilactobacillus ruminis, Acetobacter sp. 46_36, Acetobacter sp. CAG:267*	Genus: *Allobaculum;*Species: *Allobaculum mucolyticum, Allobaculum stercoricanis*
Cabana-Puig et al. (2022) [24]	In-house colony MRL/lpr mice	New MRL/lpr mice from JAX	Fecal samples (16-week-old female mice)	16S rRNA sequencing	Phylum: *Verrucomicrobia;*Family:* Porphyromonadaceae, Ruminococcaceae;*Genus: *Akkermansia, Odoribacter, Parabacteroides, Flavonifractor;*Species: *Akkermansia muciniphila*	Family: *Lachnospiraceae, Peptostreptococcaceae, Ruminococcaceae, Enterococcaceae;*Genus: *Prevotella, Coprobacillus, Turicibacter, Eubacterium, Romboutsia, Acutalibacter, Enterococcus;*Species: *Enterococcus gallinarum*

**Table 5:** Summary of studies on the human lupus nephritis microbiome. 16S rRNA = 16S ribosomal RNA; ELISA = enzyme-linked immunosorbent assay; F/B ratio = Firmicutes-to-Bacteroidetes ratio; HC = healthy controls; LN = lupus nephritis; LN.Ac = active lupus nephritis; LN.Re = lupus nephritis in remission; MLN = mesenteric lymph nodes; MRL/lpr = lupus-prone mouse strain with *Fas* mutation; MPJ = MA/MyJ mouse strain (Jackson Laboratory); N/A = not available / not applicable; PCR = polymerase chain reaction; SLE = systemic lupus erythematosus

Author (year)	Study design	Groups (number of subjects)	Microbiome source	Detection methods	Micro-organisms increased	Micro-organisms decreased
Azzouz et al. (2023) [19]	Observational	SLE patients with LN flares (n = 16); HC (n = 22)	Gut (fecal)	16S rRNA gene sequencing; shotgun metagenomic sequencing	Species: *Ruminococcus gnavus*	N/A
Cheng et al. (2025) [18]	Observational	SLE patients (n = 36: 18 SLE–non-LN, 18 SLE–LN); HC (n = 15)	Gut (fecal)	16S rRNA gene sequencing; untargeted metabolomics	Phylum: Proteobacteria ; Family: *Enterobacteriaceae*; Order: *Enterobacterales;*Class: *Gammaproteobacteria;* Genus: *Escherichia_Shigella, Bacteroides, Streptococcus, Enterococcus, Akkermansia, Lactobacillus; *Species: *Escherichia_coli*	Phylum: Firmicutes; Genus: *Faecalibacterium, Blautia*
Mohd et al. (2023) [28]	Narrative review	N/A (summarizes human and mouse studies)	Gut (fecal) - from cited studies	16S rRNA sequencing (reported from cited studies)	Genus: *Ruminococcus* (human LN, mouse), *Staphylococcus* (human LN), *Bacteroides* (mouse), *Lactobacillus* (mouse, high tryptophan diet); Species: *Ruminococcus gnavus* (human LN), *Staphylococcus aureus* (human LN), *Ruminococcus torque*s (mouse), *Bacteroides thetaiotaomicron* (mouse), *Bacteroides dorei* (mouse, high tryptophan diet), *Lactobacillus reuteri* (mouse, high tryptophan diet)	N/A
Wang et al. (2023) [16]	Systematic review	SLE-LN patients (n = 138); SLE patients (n = 441); HC (n = 1526); animal studies (n = 5)	Gut (fecal)	16S rRNA gene sequencing; metagenomic sequencing; PCR	Phylum: *Proteobacteria, Bacteroidetes;* Genus: Streptococcus (clinical), Bacteroides; Species: Ruminococcus gnavus (clinical), Ruminococcus torques (clinical and animal), Bacteroides thetaiotaomicron	Phylum: *Firmicutes* (via decreased F/B ratio); Species: *Ruminococcus torques* (animal study)
Parodi et al. (2025) [17]	Narrative review	N/A	Gut (fecal) - from cited sources	16S rRNA sequencing; metagenomic sequencing (from cited sources)	Phylum: *Proteobacteria;* Genus: *Escherichia, Prevotella;* Species: *Ruminococcus gnavus, Bacteroides thetaiotaomicron*	Phylum: F/B ratio decreased
Tan et al. (2024) [23]	Narrative review	N/A	Gut (fecal)	16S rRNA sequencing (from cited sources)	Species: *Ruminococcus gnavus*	N/A
Gui et al. (2024) [22]	Observational and experimental	SLE patients (n = 114: 62 non-LN, 52 LN); HC (n = 134); MRL/lpr mice	Gut (fecal)	16S rRNA gene sequencing; shotgun metagenomic sequencing	Genus: *Escherichia, Veillonella, Streptococcus*	N/A
Wu et al. (2025) [14]	Observational and experimental	SLE patients (n = 61: 11 non-LN, 26 active LN, 24 LN in remission); HC (n = 25); MRL/lpr mice (n = 20); MPJ mice (n = 5)	Gut (fecal); mouse spleen/MLN	16S rRNA sequencing; flow cytometry; ELISA	Phylum: *Proteobacteria;* Genus:* Escherichia-Shigella, Streptococcus, Lactobacillus, Klebsiella *(LN.Re > LN.Ac)	Genus: *Bifidobacterium* (LN.Ac most reduced), *Bacteroides, Faecalibacterium*
Lau et al. (2021) [20]	Narrative review	N/A	Gut (fecal)	16S rRNA sequencing (from cited sources)	Genus: *Lachnospiraceae, Lactobacillaceae, Bacteroides, Ruminococcus;* Species: *Ruminococcus gnavus* (strain 2, associated with severe LN)	Phylum: *Firmicutes*
Wang et al. (2022) [29]	Observational	SLE patients (n = 19); HC (n = 19)	Gut (fecal)	16S rRNA sequencing	Genus: *Streptococcus*	Genus: *Turicibacter*
Yu et al. (2022) [15]	Observational	LN patients (n = 15); HC (n = 27)	Gut (fecal)	16S rRNA sequencing	Phylum: *Proteobacteria;*Class: *Bacilli*	Phylum: *Firmicutes, Bacteroidetes;*Class: *Negativicutes, Betaproteobacteria*

Preeclampsia: Microbiome

The studies summarized in Table 6 demonstrate consistent microbiome alterations in human patients and animal models with PE, characterized by an increase in pro-inflammatory microorganisms and a decrease in beneficial microorganisms. In a 2020 case-control study by Chen et al., fecal microbiota transplantation was used to determine the relationship between the gut microbiota and the development of PE [30]. Patients with PE showed an enrichment of opportunistic bacteria, including *Fusobacterium* and *Veillonella*, while levels of beneficial bacteria, including *Faecalibacterium* and *Akkermansia*, were significantly decreased [30]. Correspondingly, levels of cytokines were markedly elevated in placental tissue from PE patients and PE-fecal microbiota transplantation mouse models [30]. Jin et al. reported a reduction in short-chain fatty acid (SCFA)-producing bacteria among patients with PE, including decreased levels of anti-inflammatory bacteria, such as *Lachnospira* and *Dialister*, and an increase in pro-inflammatory bacteria, including *Blautia* species [31]. Continued analysis revealed increased gene expression of pro-inflammatory cytokines such as IL-6 and IL-8 in early preeclampsia (EP) when compared to late preeclampsia (LP) (p = 0.005), further emphasizing the correlation between increased inflammation and the development of PE [31]. Overall, pathogenic genera, including *Fusobacterium, Veillonella, Prevotella, Bacteroides,* *Enterobacteriaceae*, and phyla Proteobacteria and Actinobacteria, increased across studies, while levels of commensal and SCFA-producing bacteria such as *Akkermansia*, *Faecalibacterium*, *Lachnospira*, *Bifidobacterium*, *Subdoligranulum*, and* Eubacterium* species decreased. The results of these studies also suggest that restoration of SCFAs may facilitate trophoblast invasion and decrease the risk of PE [31]. A summary of these microbiome changes in PE may be seen in Table 6.

**Table 6:** Microbiome changes in preeclampsia. 16S rRNA = 16S ribosomal RNA; C = control; EOPE = early-onset preeclampsia; FMT = fecal microbiota transplantation; HC = healthy controls; HDP = hypertensive disorders of pregnancy; LOPE = late-onset preeclampsia; LP = late pregnant; N/A = not available / not applicable; NP = normotensive pregnant; NP-FMT / PE-FMT = fecal microbiota transplants from normotensive or preeclamptic donors; PE = preeclampsia; qPCR = quantitative polymerase chain reaction; SCFAs = short-chain fatty acids

Author (year)	Study design	Groups (number of subjects)	Microbiome source	Detection methods	Micro-organisms increased	Micro-organisms decreased
Chen et al.(2020) [30]	Case-control	Human: PE (n = 67), NP (n = 85) Mouse PE-FMT (n = 8), NP-FMT (n = 10), controls (n = 8)	Gut (fecal)	16S rRNA gene sequencing; qPCR in situ hybridization	Genus: *Fusobacterium, Veillonella*	Genus: *Faecalibacterium, Akkermansia*
Jin et al. (2022) [31]	Case-control	Human: PE (n = 35); healthy pregnant controls (n = 35)	Gut (fecal)	16S rRNA gene sequencing	Phylum: *Actinobacteria, Cyanobacteria, Fusobacterium*	Phylum: *Verrucomicrobia;*Genus: *Akkermansia*
Tian et al. (2024) [32]	Case-control	EOPE (n = 48); LOPE (n = 32); HC (n = 96)	Gut (fecal)	16S rRNA gene sequencing	Species: *Clostridium butyricum;* Genus: *Dialister, Veillonella, Fusobacterium*	Genus: *Lachnospira, Akkermansia, Faecalibacterium*
Beckers et al. (2020) [33]	Narrative review	N/A	Gut, placental, oral, vaginal	16S rRNA gene sequencing; qPCR	Phylum: *Proteobacteria;*Genus: *Fusobacterium, Prevotella, Bacteroides, Lactobacillus*	Genus: *Faecalibacterium, Akkermansia, Bifidobacterium, Lachnospira*
Ishimwe, JA (2021) [34]	Narrative review	N/A	Gut, placental, oral, vaginal	16S rRNA gene sequencing	Species: *Helicobacter pylori;*Phylum: *Actinobacteria, Proteobacteria;*Genus: *Bacteroides*	Phylum: *Firmicutes*
Giugliano et al. (2025) [35]	Case-control	PE (n = 30); normotensive controls (n = 25)	Gut (fecal)	16S rRNA gene sequencing	Order: *Clostridiales*	Phylum: *Firmicutes;*Genus: *Bacteroides, Lachnospiraceae, Ruminococcaceae*
Lv et al. (2024) [36]	Case-control	PE (n = 19); normotensive controls (n = 20)	Gut (fecal)	16S rRNA sequencing; shotgun metagenomic sequencing	Genus: *Blautia, Bilophila, Fusobacterium, Ruminococcus*	Species: *Alistipes shahii;*Genus: *Bacteroides, Phocaeicola, Parabacteroides, Akkermansia, Dialister, Faecalibacterium, Gemmiger, Methanobrevibacter*
Han et al. (2025) [37]	Case-control	PE (n = 34); LP control (n = 39)	Gut (fecal)	16S rRNA gene sequencing	Genus: *Prevotella, Erysipelotrichaceae_UCG‑003, Dorea*	Genus: *Subdoligranulum, Parabacteroides, Bacteroides*
Lin et al. (2020) [38]	Case-control	Primary cohort: SPE (n = 30) vs controls (n = 30); Confirmation cohort: SPE (n = 580) vs controls (n = 55)	Vaginal	16S rRNA gene sequencing; qPCR	Species: *Prevotella bivia;*Genus: *Atopobium, Aerococcus*	N/A
Wu et al. (2024) [39]	Case-control	HDP patients (n = 6); normotensive controls (n = 9)	Gut (fecal)	16S rRNA gene sequencing; bioinformatics	Species: *E. coli;*Genus: *Rothia, Actinomyces, Enterococcus*	Genus: *Coprococcus*
Chang et al. (2020) [40]	Case-control	Severe PE patients (n = 27); healthy controls (n = 36)	Gut (fecal)	16S rRNA gene sequencing; gas chromatography for SCFAs	Phylum: *Proteobacteria;*Genus: *Enterobacter, Escherichia_Shigella*	Species: *Eubacterium_rectale, Eubacterium_hallii;* Genus: *Blautia, Streptococcus, Bifidobacterium, Collinsella, Alistipes, Subdoligranulum*

Discussion

Lupus Nephritis: Alpha Diversity

Across the included studies, a consistent reduction in gut microbiome alpha diversity was observed in LN patients compared with healthy controls or SLE patients without nephritis. Wu et al. assessed alpha diversity using the Chao1 and Simpson indices, both showing significantly reduced diversity in LN patients [14]. Gui et al. evaluated alpha diversity using observed operational taxonomic units (OTUs) and the SI, reporting significantly lower richness and diversity in SLE patients, with more pronounced reductions in those with LN [22]. Wang et al. assessed alpha diversity using observed OTUs, Chao1, and the SI, all of which were significantly reduced in SLE patients, though the equitability index did not differ significantly between groups, indicating preserved evenness despite reduced richness [29]. Yu et al. reported significantly reduced alpha diversity using observed OTUs, Shannon, and Chao1 indices in LN patients compared to healthy controls [15]. Additionally, the systematic review by Wang et al. and the review article by Parodi et al. summarized consistent evidence of decreased alpha diversity across LN studies [16,17]. Cabana-Puig et al. also demonstrated comparable reductions in alpha diversity in lupus-prone mice [24]. In contrast, Cheng et al. found no significant differences in alpha diversity between LN patients, SLE patients without nephritis, and healthy controls [18].

Lupus Nephritis: Beta Diversity

Across the included studies, significant differences in beta diversity were also observed. Azzouz et al., Wang et al., Cheng et al., and Gui et al. reported distinct clustering patterns or greater variability in microbial community composition among LN patients compared to healthy controls or SLE patients without nephritis [18,19,22,29].

Lupus Nephritis: Microbiome Findings

Several prior studies have identified a reduced F/B ratio as a hallmark of gut dysbiosis in SLE when compared to healthy controls, with a lower ratio associated with increased disease activity [41-43]. This pattern has also been noted in other inflammatory diseases such as rheumatoid arthritis [44]. However, whether a reduced F/B ratio is consistently present in LN remains unclear. In the observational studies reviewed here, we did not consistently observe a reduced F/B ratio among LN patients, contrasting with findings more commonly described in narrative reviews and highlighted in one systematic review [16]. Our analysis yielded three main findings: increased abundance of *Streptococcus*,* E. coli*, and *R. gnavus* in LN patients. Additional research is needed to clarify the role of the F/B ratio in LN, as it is possible that this ratio might shift during progression from SLE to SLE-LN. Additionally, it is important to consider that these studies may have prioritized microbial taxa that directly contribute to LN pathogenesis rather than broad metrics such as the F/B ratio. For example, the lack of reduced F/B ratio reported by Gui et al. may reflect a subset of SLE-LN patients with distinct microbial profiles [22]. The prominence of *R. gnavus* and other specific taxa suggests that SLE-LN may have a unique microbial pattern that amplifies renal inflammation, potentially extending beyond the systemic dysbiosis noted in SLE. The limited reporting of the F/B ratio in primary LN studies emphasizes the need for additional research directly comparing the microbiome between SLE without nephritis and SLE-LN.

Preeclampsia: Alpha and Beta Diversity

A study conducted by Tian et al. analyzed the gut microbiome of early-onset PE (EOPE), late-onset PE (LOPE), and healthy pregnant controls (HPC) using 16S rRNA gene sequencing [32]. EOPE was associated with reduced alpha diversity (α-diversity) and depletion of SCFA-producing bacteria, which may contribute to the endothelial and placental dysfunction observed in PE patients [32]. A 2025 study by Han et al. analyzed the diversity and overall composition of the gut microbiota in PE patients by using fecal samples [37]. PE patients showed significant differences in beta diversity (β-diversity) when compared to the control group, while α-diversity did not differ significantly. More specifically, levels of *Prevotella*,* Dorea*, and Erysipelotrichaceae_UCG-003 were significantly increased in the PE group compared to the control group, while *Subdoligranulum*,* Parabacteroides*, and *Bacteroides* were significantly decreased [37]. Although α-diversity findings vary across studies, reductions have been associated with notable dysbiosis in PE [32,39].

Preeclampsia: Short-Chain Fatty Acids and Their Impact on the Microbiome

SCFAs are short-chain metabolic byproducts produced through the gut and have been associated with the development of hypertension [45]. Among various SCFAs, the three found in the highest concentrations in the gut include acetic acid (acetate), propionic acid (propionate), and butyric acid (butyrate) [45]. Studies have shown that patients with PE exhibit lower levels of SCFAs, which may contribute to an increased inflammatory state [46]. It has been hypothesized that SCFAs are able to modify blood pressure and inflammation through binding to secondary messenger systems; more specifically, to G protein-coupled receptors (GPCRs) expressed in renal tissue [45]. By binding to GPCRs and modulating the NF-κB signaling pathway, higher levels of SCFAs promote increased downstream production of T-regulatory cells (T regs) and a decreased state of inflammation [45]. Jin et al. found that treating PE-model rats with a propionate-producing bacterium such as *Akkermansia muciniphila *led to decreased blood pressure and improved placental outcomes [31]. The variability of SCFA content found in patients with PE may be of diagnostic value when used in early screening for PE. A study by Li et al. found significantly lower levels of caproic acid and butyrate in women with PE when compared to healthy controls [47].

Lupus Nephritis: Short-Chain Fatty Acids Impact on the Microbiome

Narrative reviews by Mohd et al. and Parodi et al., as well as a systematic review by Wang et al., report that gut microbiota dysbiosis in SLE patients is associated with increased fecal SCFA levels and a reduced F/B ratio [16,17,28]. These reviews suggest that imbalances in SCFAs may contribute to SLE pathogenesis by promoting a pro-inflammatory, dysbiotic state. In SLE patients, particularly those with LN, Tan et al. noted that reduced levels of SCFA-producing bacteria, including *Faecalibacterium* and *Roseburia,* lead to lower SCFA levels, which may disrupt regulatory T-cell function and elevate pro-inflammatory cytokines such as IL-6 [23]. This shift may potentially exacerbate kidney damage through increased gut permeability. Similarly, Wu et al. demonstrated in a mixed human-animal study that SCFA supplementation through *Bifidobacterium-*derived metabolites restored immune balance and mitigated kidney damage in lupus-prone mice, suggesting a potential protective role for SCFAs [14]. Collectively, these findings regarding SCFAs further emphasize the need for primary research directly comparing the microbiome role in SLE-non LN versus LN. SCFAs may play a role in the progression from SLE to LN, and supplementation could offer therapeutic benefits.

Overlap and Distinguishing Characteristics

This review reveals both shared and distinct gut microbiome features in LN and PE. Enrichment of Proteobacteria (5/11 LN studies; 3/10 PE studies) and *Escherichia* (4/11 LN; 2/10 PE) reflects a shared pro-inflammatory dysbiosis, as Proteobacteria expansion can disrupt gut barrier integrity, increase intestinal permeability, and promote systemic inflammation with renal consequences [48]. Both conditions demonstrate reduced *Faecalibacterium* (2/11 LN; 4/10 PE) and a less consistent depletion of *Bifidobacterium* (reported in LN by Wu et al. and in PE by Chang et al.), suggesting that the loss of anti-inflammatory taxa may further exacerbate inflammation [39,40].

Distinct microbial patterns were found to be disease-specific: *Ruminococcus gnavus (R. gnavus) *correlated with LN severity, potentially by promoting autoantibody generation or immune over-activation, while *Fusobacterium* enrichment and *Akkermansia* depletion in PE may reflect pregnancy-specific inflammation or placental dysfunction [49]. LN also demonstrated increased *Streptococcus*, whose antigens may trigger molecular mimicry and cross-reactive autoantibody production, further contributing to renal injury [50]. These findings suggest therapeutic potential for microbiome modulation. Restoring *Faecalibacterium* and *Bifidobacterium* through probiotics or high-fiber diets may benefit both diseases; while targeting *R. gnavus* may be more relevant to LN, and reducing *Fusobacterium*, via diet or vaginal probiotics, may be more pertinent to PE. Moreover, these taxa hold promise as biomarkers, with *R. gnavus* linked to LN severity and *Fusobacterium* associated with PE.

Beyond the gut, Monticolo et al. identified *Ureaplasma urealyticum* and *Ureaplasma parvum* in the vaginal microbiota of SLE patients, though these findings may have been confounded by concurrent steroid or antibiotic use; importantly, data on genital dysbiosis in LN remain limited [51]. Shi et al. further demonstrated reduced urinary microbiome diversity and identified potential urinary biomarkers in SLE [52]. In PE, interest has expanded to maternal microbial niches, including the gut, vagina, oral cavity, and placenta, where enrichment of *Bacteroides*,* Fusobacterium*, and *Prevotella*,* *and depletion of *Lactobacillus*, *Faecalibacterium*, and *Bifidobacterium* have been reported [38-40,53]. These microbial shifts are associated with increased lipopolysaccharide burden, heightened Toll-like receptor signaling, systemic inflammation, and reduced SCFA production, while microbial metabolites such as SCFAs, trimethylamine N-oxide, and bile acids may further influence endothelial and placental function [31,40].

Limitations and Future Directions

Future research can take several directions to address key gaps and advance this comparative work. For SLE-LN, the inconsistent findings regarding the F/B ratio highlight the need for studies directly comparing SLE patients with and without nephritis. Additional research is needed for further characterization of microbiome findings in EOPE and LOPE, as well as to explore how differing dietary and nutritional factors influence microbiome composition.

One limitation of this review includes the comparison of findings across varying sample types. For example, microbial samples were derived from gut, vaginal, and placental microbiomes, which differ significantly in baseline microbial composition. Moreover, microbial detection methods were not standardized across studies and included techniques such as 16S rRNA sequencing, gas chromatography, shotgun metagenomic sequencing, and polymerase chain reaction. Future research directions may benefit from focusing on specific microbiome sample types and comparing the content of organisms found in each.

Recent research has begun to explore the role of the gut and urinary microbiome in modulating immune responses in SLE and its renal manifestation, LN. Microbial dysbiosis, particularly in the gut, may contribute to both disease initiation and progression, offering a novel lens through which to understand autoimmunity. This emerging insight has opened new avenues for examining host-microbiome interactions not only as potential biomarkers but also as therapeutic targets in LN and other inflammatory renal conditions, such as PE. However, unlike PE, which arises primarily from placental dysfunction, LN develops from systemic autoimmunity. Multiple studies in our review demonstrated that patients with SLE and LN exhibit significant gut dysbiosis, characterized by reduced abundance of immunoregulatory taxa such as *Faecalibacterium* and increased levels of pro-inflammatory bacteria, including *R. gnavus,* *Enterococcus*, and other pathobionts [14,18,19,21,22].

Lupus Nephritis: Possible Therapeutic Implications

Azzouz et al. reported an increased abundance of *R. gnavus* in nearly half of patients experiencing LN disease flares [19]. This expansion was accompanied by the expression of a novel lipoglycan recognized by elevated immunoglobulin G2 (IgG2) antibody levels, suggesting that *R. gnavus *may serve as a potential biomarker for disease monitoring and a target for further therapeutic inventions [19]. 

Additionally, Cheng et al. identified distinct bile acid profiles in SLE patients with and without nephritis [18]. Elevated levels of Glycocholic acid and Glycochenodeoxycholic acid were observed in SLE-non LN patients, whereas reduced bile acid levels and increased Mead acid levels were noted in SLE-LN patients [18]. A negative correlation between *Escherichia-Shigella* abundance and bile acid concentrations was also reported. These metabolic alterations suggest dysfunction of the gut-kidney axis in LN, highlighting a potential role for dietary modulation in managing renal inflammation. Zhu et al. further explored the impact of synbiotics on gut microbiota in new-onset LN patients through metagenomic and metabolomic sequencing [54]. Following administration of synbiotics in addition to prednisone acetate and cyclophosphamide, analysis of patients’ fecal samples revealed a decrease in pathogenic bacteria, including *Prevotella*, *Bacteroides*, and Enterobacteriaceae unclassified, and an increase in Actinobacteria and Firmicutes [54]. Corresponding alterations in metabolic pathways, such as amino acid biosynthesis and purine metabolism, were observed and correlated with clinical data. These findings suggest that synbiotics may serve as a promising adjunctive therapy for LN.

Preeclampsia: Possible Therapeutic Implications

A 2021 study analyzing various maternal microbiome sites, including placental, vaginal, and gut samples, demonstrated increased dysbiosis among patients with PE [34]. Probiotics, prebiotics, or nutritional modification may serve as new therapeutic strategies for PE by combating this dysbiosis and restoring physiologic homeostasis [34]. Patients with PE consistently exhibit reduced fecal microbiome diversity. Notably, fecal levels of butyrate were significantly decreased in PE, whereas exogenous butyrate supplementation significantly lowered blood pressure in the LPS-induced hypertensive rat models [40]. This correlation supports the potential role of dietary modifications aimed at increasing butyrate levels as a treatment approach for patients with PE [40]. Similarly, a study by Sun et al. concluded that PE rat models treated with probiotics experienced increased levels of *Bifidobacterium, Lactobacillus*, and nitric oxide (p < 0.05), as well as normalized blood pressure compared to untreated controls [53]. These findings further highlight the potential benefits of probiotic-mediated microbiome restoration. Additional studies have highlighted the role of an overactive innate immune system in PE, particularly through the Toll-like-receptor 4 system signaling pathway, which promotes the secretion of inflammatory cytokines and inhibits effective trophoblast invasion [55]. Management approaches that attenuate Toll-like-receptor-mediated inflammation may help reduce PE severity.

Restoring SCFA levels in patients with PE may support trophoblast implantation and outweigh negative microbiome changes associated with the disease. Additionally, vitamin D has recently emerged as a potential modulator of the microbiome. Lower levels of vitamin D have been associated with PE, and a 2023 study by Ma et al. showed that vitamin D supplementation reduced inflammation and increased SCFA-producing bacteria in the microbiome [37,55].

## Conclusions

This study underscores the critical role of a pro-inflammatory microbiome in the pathogenesis of both PE and LN. Shared microbial features, such as elevated *Proteobacteria* and *Escherichia*, suggest dysbiosis as a common driver of inflammation, while disease-specific alterations, including *Fusobacterium* enrichment and *Akkermansia* depletion in PE, and* Ruminococcus gnavus* expansion in LN, highlight distinct mechanisms. These microbial shifts contribute to systemic inflammation, endothelial dysfunction, immune dysregulation, and organ damage, converging on inflammatory pathways characterized by elevated tumor necrosis factor-α, interleukin-6, and related cytokines. The identification of both shared and unique microbial signatures offers opportunities for developing diagnostic biomarkers, targeted microbiome-modulating therapies, and personalized treatment strategies. Future longitudinal and interventional studies are needed to clarify causal relationships and determine whether modifying the microbiome can improve maternal-fetal outcomes in PE or mitigate renal injury in LN. Such insights may pave the way for precision medicine approaches in both obstetric and autoimmune disease contexts.
